# Stepped-care to prevent depression and anxiety in visually impaired older adults – design of a randomised controlled trial

**DOI:** 10.1186/1471-244X-13-209

**Published:** 2013-08-09

**Authors:** Hilde PA van der Aa, Ger HMB van Rens, Hannie C Comijs, Judith E Bosmans, Tom H Margrain, Ruth MA van Nispen

**Affiliations:** 1 Department of Ophthalmology, VU University Medical Centre, P.O. Box 7057, Amsterdam, MB, 1007, The Netherlands; 2 EMGO Institute for Health and Care Research (EMGO+), VU University Medical Centre, Van der Boechorststraat 7, Amsterdam, BT, 1081, The Netherlands; 3 Department of Ophthalmology, Elkerliek Hospital, Wesselmanlaan 25, Helmond, HA, 5707, The Netherlands; 4 Department Psychiatry VUmc/ GGZinGeest, A.J.Ernststraat 1187, Amsterdam, HL, 1081, The Netherlands; 5 Department of Health Sciences and EMGO Institute for Health and Care Research, Faculty of Earth and Life Sciences, VU University Amsterdam, Amsterdam, The Netherlands; 6 School of Optometry and Vision Sciences, Cardiff University, CF24, Cardiff, 4LU, United Kingdom

**Keywords:** Low vision, Stepped-care, Older adults, Depression, Anxiety, Prevention

## Abstract

**Background:**

Subthreshold depression and anxiety are common in the growing population of visually impaired older adults and increase the risk of full-blown depressive or anxiety disorders. Adequate treatment may prevent the development of depression or anxiety in this high risk group.

**Method/design:**

A stepped-care programme was developed based on other effective interventions and focus groups with professionals and patient representatives of three low vision rehabilitation organisations in the Netherlands and Belgium. The final programme consists of four steps: 1) watchful waiting, 2) guided self-help, 3) problem solving treatment, 4) referral to general practitioner. The (cost-)effectiveness of this programme is evaluated in a randomised controlled trial. Patients (N = 230) are randomly assigned to either a treatment group (stepped-care) or a control group (usual care). The primary outcome is the incidence of depressive and anxiety disorders, measured with the Mini International Neuropsychiatric Interview (MINI).

**Discussion:**

Preventive interventions for depression and anxiety have received little attention in the field of low vision. A stepped-care programme that focuses on both depression and anxiety has never been investigated in visually impaired older adults before. If the intervention is shown to be effective, this study will result in an evidence based treatment programme to prevent depression or anxiety in patients from low vision rehabilitation organisations. The pragmatic design of the study greatly enhances the generalisability of the results. However, a possible limitation is the difficulty to investigate the contribution of each individual step.

**Trial registration:**

Identifier: NTR3296

## Background

According to the World Health Organisation, 285 million people worldwide are visually impaired, of which 65% is 50 years or older [1]. Due to an aging population the prevalence of visual impairment in developed countries will only increase in the future. In 2008, 311,000 people in the Netherlands were visually impaired. It is estimated that in 2020 this number will increase by 18% to 367,000 people [2].

Depression and anxiety symptoms are common in visually impaired older adults. Recent studies suggested that approximately one-third (range 22-42%) of visually impaired older adults experience mild but clinically significant depressive or anxiety symptoms, also known as subthreshold depression or anxiety [3-6]. This is at least twice as high as the prevalence in the general population (10-15%) [7]. It is important to treat symptoms of depression and anxiety in an early stage, because these are the most important predictors of developing a full-blown depressive or anxiety disorder according to DSM-V criteria, such as major depression, phobic disorders or generalized anxiety disorder [8]. Studies suggested that loss of vision increases the risk of depressive disorders [9,10].

Depressive and anxiety disorders decrease quality of life, the ability to cope with daily life activities and even life expectancy [11]. Even subthreshold manifestations of depression and anxiety already have adverse consequences for quality of life [7,12] and increase health care utilisation [7]. Depression and anxiety often accompany disabling diseases and aggravate existing disability [13]. In addition, they may influence factors that are necessary for successful rehabilitation, such as the ability to learn new tasks, processing information and being oriented towards achieving certain goals [5,14]. Depression and anxiety (both threshold and subthreshold) often occur together, which causes even more disability and distress in daily life [15-17]. Research has shown that very few older adults experience only depressive or anxiety disorders, without at least some symptoms of the other [16,18].

Some studies have shown that interventions aimed at preventing major depression and/or reducing subthreshold symptoms of depression in the visually impaired can be effective. Horowitz et al. (2005) found that variable low vision rehabilitation services, such as counselling and use of optical devices had a small positive effect on the decline of depressive symptoms after two years [5,19]. In another trial, with a follow-up period of six months, Horowitz et al. (2006) showed that optical devices that optimise residual vision, as apposed to adaptive aids that involve learning new methods to compensate for lost functions, had a positive effect on the course of depressive symptoms [20]. Brody et al. (2006) found that a self-management programme consisting of cognitive and behavioural elements including health education and enhancement of problem-solving skills, significantly reduced depressive symptoms in people with age-related macular degeneration (AMD) after six months [21]. Rees et al. (2007) concluded that self-management programmes for visually impaired adults are a promising way to help address emotional distress [22]. Girdler et al. (2010) also evaluated a self-management programme in visually impaired older adults and reported significantly less depressive symptoms at 12 weeks in participants who received the programme as apposed to patients who received standard visual rehabilitation services [23]. Rovner and Casten (2008) found that problem solving treatment (PST), a short behavioural treatment in which participants learn a new method to address problems that interfere with everyday functioning, prevented the onset of depressive disorders in elderly with AMD after two months. However, after six months there was no statistically significant difference in depressive disorders between the intervention- and control group [24]. To prevent the onset of depression on the long term Rovner and Casten (2008) suggest to either continue treatment in this high risk group after providing PST or focus on preventive treatment for patients that show early signs of depression [24]. Currently, Rovner and colleagues are performing the Improving Function in Age-related Macular Degeneration study (IF-AMD) in which they are investigating the efficacy of PST compared to Supportive Therapy in preventing depressive disorders [25]. Margrain and colleagues are currently performing the Depression in Visual Impairment Trial (DEPVIT) to reduce depressive symptoms in visually impaired adults. They are comparing three groups: one group that receives PST with additional self-help materials, one group that is referred to the general practitioner (GP) and one ‘waiting list control’ group [26].

These studies together suggest that low vision rehabilitation services, self-management programmes and PST can be effective in addressing depressive symptoms and depressive disorders among visually impaired people. However, only a few studies evaluated the effectiveness of such interventions and these studies were only focused on depression and not on anxiety. Moreover, economic evaluations of such interventions are completely missing.

This project aims to design and test the (cost-)effectiveness of a stepped-care programme to prevent the onset of depressive and anxiety disorders in visually impaired older adults (50 years and older) with subthreshold depression and/or anxiety, in three low vision rehabilitation organisations in the Netherlands and Belgium. By reducing symptoms of depression and anxiety, the intervention is expected to positively influence (vision-related) quality of life and adaptation to vision loss. It is an indicated preventive intervention, aimed at persons who show early signs of depression and/or anxiety but do not meet the diagnostic criteria. The aim is to prevent or delay the onset of major disorders and to reduce the severity and shorten the duration of existing symptoms. Stepped-care comprises different treatment components, such as self-help and PST. The general idea is that if the first, less intensive step does not lead to a reduction of symptoms, then a patient moves to a next step which consists of a more intensive and expensive treatment type. This type of intervention is expected to be efficient, because not all patients need the same type or intensity of treatment [27]. Several randomised controlled trials (RCTs) outside the field of low vision found that a stepped-care intervention can be effective in addressing depression and/or anxiety [28-33]. Furthermore, both the Multidisciplinary guidelines for mental healthcare in the Netherlands as the National Institute for Health and Clinical Excellence (NICE) in the United Kingdom recommend using a stepped-care model to address depression in older adults [34,35].

## Method/design

### Development of a stepped-care programme for visually impaired older adults

#### Evidence from previous studies

The first step in developing a new stepped-care programme for visually impaired older adults was to search the literature for other stepped-care programmes and interventions that were found to be effective in addressing depression and anxiety.

Different RCTs outside the field of low vision found that stepped-care programmes can be effective in reducing depression and anxiety. Araya et al (2003) found that a stepped-care programme for low-income women in Santiago (Chile), consisting of a psycho-educational group intervention and drug treatment, was effective in reducing depression after 6 months [28]. Davidson et al. (2010) found that a stepped-care programme, consisting of PST and psychotherapy or medication, significantly decreased depressive symptoms in patients with acute coronary syndrome (ASC) and even improved the ASC prognosis [29]. Patel et al. (2010) showed that a collaborative stepped-care programme, consisting of psycho-education, psychotherapy and medication, was effective in reducing depressive and anxiety disorders in primary care [30]. Unutzer et al. (2008) found that a collaborative stepped-care programme, consisting of PST and medication, was cost-effective in reducing late-life depression in primary care after 12 months, with total health care cost calculations over a 4-year period [31]. Van ‘t Veer et al. (2009) showed that a stepped-care programme for older adults in general practice (75 years and older) was effective in preventing depression and anxiety disorders after 12 months [32], with effects sustaining even after 24 months [33]. This programme consisted of a period of watchful waiting, bibliotherapy (the use of reading materials as guidance), PST and referral to the GP.

A recent systematic review concluded that cognitive behavioural therapy (CBT) and PST can prevent the development of depression and anxiety in patients with subclinical manifestations of these disorders [36]. Both treatment types help patients to acknowledge their symptoms and encourage them to switch to more active self-management strategies [36]. Studies in the field of low vision suggest that rehabilitation services, self-management programmes and PST can be effective in addressing depressive symptoms and depressive disorders among visually impaired people [19-24]. Based on these findings an initial protocol of the stepped-care programme for this study was developed.

#### Focus groups

The second step in developing the protocol was to involve health care workers and patient representatives of the rehabilitation organisations. In this qualitative phase of the study, the initial protocol was discussed in one focus group with 12 low vision health care workers (social workers and psychologists) and two focus groups with each 4 patient representatives (one in the Netherlands and one in Belgium). Different aspects of the stepped-care programme were discussed: intensity of guidance, type of health care workers within rehabilitation organisations who could offer guidance, accessibility of the intervention and ways to stimulate patients to participate. Based on these group meetings the initial protocol was adjusted. After the adjustments, the protocol was sent to the same health care workers and patient representatives. After some revisions, the final content of the protocol was determined.

#### Final protocol

**Figure 1:**
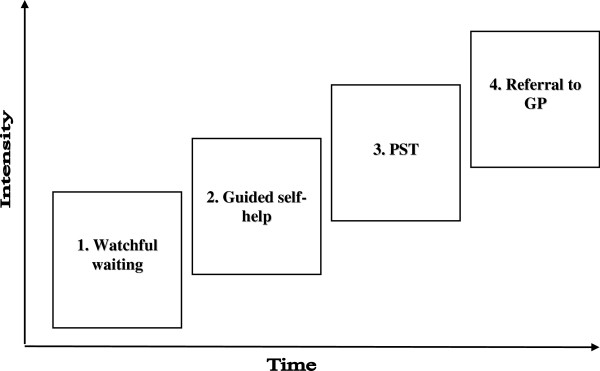
Stepped-care programme.

Figure 1 shows the final stepped-care programme, which consists of four consecutive steps. Each step takes approximately three months. Only if symptoms of depression and/or anxiety persist, then patients move on to the next step. Severity of symptoms is measured with the Epidemiologic Studies Depression scale (CES-D) [37,38] and the Hospital Anxiety and Depression Scale – Anxiety (HADS-A) [39]. Only if patients have a score of 16 or higher on the CES-D and/or a score of 7 or higher on the HADS-A, then they move on to the next step.

#### Step 1: watchful waiting

In the first step, patients are followed to see if symptoms of depression and/or anxiety recover spontaneously. Patients are contacted by one of the researchers (first author) at the beginning of this step to discuss their problems of depression and/or anxiety and give an explanation of this first step of the programme. Patients are told that they can contact the researcher during this period if necessary, for example if symptoms of depression and/or anxiety get worse or questions arise. In several studies it was shown that symptoms of depression and anxiety may recover spontaneously in this first period [36,40-42].

#### Step 2: guided self-help

When patients still have elevated scores on the CES-D or HADS-A after 3 months, they receive the self-help course “Glance at your Dip” (‘Blik op je Dip’ in Dutch). This course is based on the course of Cuijpers et al. (2003) (called ‘In de put, uit de put’ in Dutch) which was found to be highly effective in preventing depressive and anxiety disorders [43,44]. Techniques of CBT and PST are used in this course.

Based on the focus groups, the self-help course was rewritten and adapted specifically for patients with visual impairments, e.g. by adding examples of problems people often encounter in daily life due to their visual impairment. The self-help course is offered in a written (large font), a digital, an audio and Braille version. The self-help course helps patients to address depressogenic and anxiogenic thinking, improve social skills and increase pleasant activities and relaxation. The aim is to help patients to cope better with problems related to one’s symptoms of depression and/or anxiety, such as vision loss.

Guidance in following the self-help course is provided by occupational therapists who received a one-day training (by the first and last author). Training consisted of information about depression and anxiety, an explanation of the stepped-care programme in total, the self-help course and techniques to motivate patients in addressing their problems.

In this second step of the programme, the trained occupational therapist contacts the patient by telephone and introduces the course. If the patient agrees, two face-to-face contacts (at the rehabilitation organisation or at home) take place. Subsequently, the occupational therapist conducts several telephone calls and may schedule another face-to-face contact to encourage patients to continue with the course. The aim of the contacts is 1) to explore the symptoms of depression and anxiety, 2) to reflect on these symptoms by exploring everyday problems, 3) to create awareness of the problems and the possibility to address them, 4) to encourage participants to complete/continue the self-help course. The first two goals help to establish a working relationship between the patient and the occupational therapist. The second two goals help patients recognise their problems and the possibility to address them even in an early stage of the complaints.

Older adults who feel depressed often find it difficult to find the energy and motivation to work on their problems. Therefore, techniques of Motivational Interviewing (express empathy, roll with resistance not fight it, support self-efficacy) are used to stimulate this. Research shows that these techniques can be effective in stimulating patients in seeking and accepting help and making changes in their behaviour [45].

The researchers (first author; social worker and last author; psychologist), who developed the self-help course, monitor the execution of the intervention by keeping in close contact with the occupational therapists by telephone and e-mail (at least one contact every two weeks) and by organising a peer group meeting to share experiences and learn from each other. Additionally, occupational therapists and patients are asked to evaluate the self-help course on a written evaluation form. They are asked what they think about the intervention, about the guidance that was given and about the results.

#### Step 3: problem solving treatment (PST)

When patients still have elevated scores on the CES-D or HADS-A after step 2, they receive PST by a trained social worker or psychologist at the rehabilitation organisation. PST is a short evidence-based behavioural treatment which helps patients to regain control of their life, which reduces feelings of depression and anxiety [46]. With PST, the patient learns to identify and address problems that interfere with everyday functioning and lead to feelings of depression and anxiety by means of 7 different steps: 1) clarifying the problem, 2) establishing a realistic goal to address the problem, 3) generating multiple solution alternatives, 4) exploring the pros and cons for each possible solution, 5) choosing the preferred solution, 6) identifying the specific steps needed to carry out the solution, 7) after trying to implement the solution, in the next meeting the process will be evaluated.

Social workers and psychologists received a one-day training in two groups by a qualified PST trainer and supervisor (psychologist, PhD) with experience in stepped-care. Information about the techniques and different steps of PST was given and was practiced in role-plays. After that, all social workers and psychologists piloted their learned skills on a patient that did not participate in this study.

In this third step of the programme the social worker or psychologist contacts the patient by telephone and introduces PST. If the patient agrees, subsequently a maximum of seven face-to-face contacts (at the rehabilitation organisation or at home) take place in which the stages of PST are explained and applied to problems encountered in daily life. Patients choose their own problems to work on and are able to use a list (large font) of potential problems (related to physical-, psychological-, practical- and social functioning) to help them think of problems to address. Furthermore, a CD with the different steps of PST is given to patients as a reminder for practising at home in between the meetings.

To secure fidelity with the intervention, a qualified PST trainer and supervisor (psychologist, PhD) guides social workers and psychologists in performing PST, by means of telephone calls and e-mail. In addition, a peer group meeting is organised to share experiences and learn from each other. Two sessions per patient are audio-taped to have a good understanding of the process. The supervisor and researcher (first author) review these tapes. Additionally, social workers, psychologists and patients are asked to evaluate the process. They are asked what they think about the intervention, about the guidance that was given and about the results.

#### Step 4: referral to general practitioner (GP)

When symptoms persist, patients are referred to their GP to discuss further (more intensive) treatment and to discuss the use of medication. GPs are the so called ‘gate-keepers’ for all (mental) health care facilities in the Netherlands. They can refer patients to other care providers or may prescribe medication. If patients are diagnosed with a depressive or anxiety disorder during the study, as measured with the Mini International Neuropsychiatric Interview (MINI) [47], they are directly referred to their GP to discuss further treatment and medication.

Not all patients in the intervention group complete all phases of the stepped-care intervention, because treatment is only initiated if symptoms of depression and/or anxiety remain elevated. Patients who recover enter a period of ‘watchful waiting’. When elevated symptom levels again indicate a need for treatment, the following step of the intervention is initiated.

### Design of a randomised controlled trial (RCT)

The design of the study is a two-armed multicentre international RCT, conducted at two rehabilitation organisations in the Netherlands and one in Belgium, to evaluate the (cost-) effectiveness of the new stepped-care programme to prevent depression and anxiety in visually impaired older adults. The study protocol was approved by the Medical Ethics Committee of the VU University Medical Centre in Amsterdam, the Netherlands and the University Hospital Leuven in Belgium. It is conducted according to the principles of the Declaration of Helsinki. Patients are fully informed about the study and give written informed consent. They are allowed to withdraw this consent at any time during the study.

#### Setting

Low vision rehabilitation organisations in the Netherlands and Belgium operate at the interface between health care and social care. They support people with vision loss by training them to make use of their residual vision and cope with everyday problems aimed at home-, leisure-, school-, work or other activities and participation issues. That is why a programme to address depression and anxiety, in addition to usual visual rehabilitation care, fits well within this setting.

#### Recruitment

The invitation of patients (approximately N = 3000) is done in four waves, with three months in between (September 2012, December 2012, March 2013 and June 2013). This is to avoid the need for all patients having to be treated at the same time. Patients from the low vision rehabilitation organisations who are 50 years or older receive an information letter and an informed consent form. After they give written consent to participate, they are screened for eligibility.

#### Participants

Visually impaired older adults with subthreshold depression or anxiety, but no actual depression or anxiety disorder according to the DSM IV, with sufficient knowledge of the Dutch language, without severely impaired cognitive functioning and capable to give informed consent are eligible to participate. Depressive symptoms are measured with the Centre for Epidemiologic Studies Depression scale (CES-D) [37,38] and anxiety symptoms with the Hospital Anxiety and Depression Scale – Anxiety (HADS-A) [39]. Visually impaired older adults who have a score of 16 or higher on the CES-D or a score of 7 or higher on the HADS-A are eligible to participate. If participants have a depressive or anxiety disorders according to the DSM IV as measured with the Mini International Neuropsychiatric Interview (MINI) they are excluded from the study [47]. Cognitive functioning is evaluated by means of a six-item screener. This is a modified version of the Mini-mental state examination (MMSE), with a score of 3 or more errors indicating cognitive problems [48]. Patients who have no complaints and patients with a depressive or anxiety disorder after the first screening are not randomised but are followed in this study to see how symptoms develop over time.

#### Randomisation

Patients are randomly assigned to either the intervention group (stepped-care programme) or the control group (usual care). Randomisation is performed using a computer-generated allocation scheme based on blocks of two, stratified by 17 different locations of the three low vision rehabilitation organisations in both the Netherlands and Belgium. Usual care consists of the support low vision rehabilitation organisations normally offer to visually impaired older adults and/or care that is received by other providers on the initiative of patients.

#### Masking

Masking of participants and therapists is not possible due to the nature of the intervention. However, the principal investigator and the research assistants are masked until after the primary outcomes of the study are analysed.

#### Measurements

**Figure 2:**
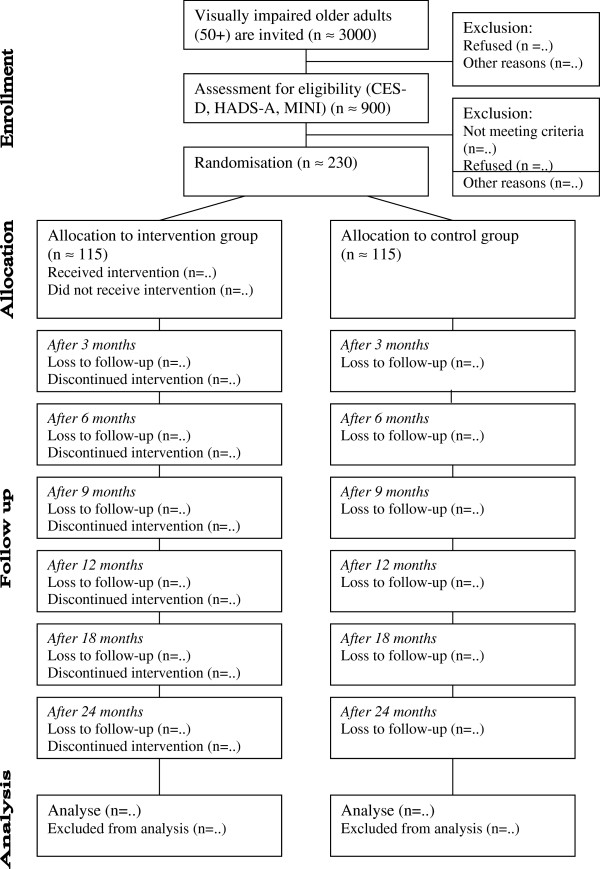
Overview of the RCT design.

In total seven measurements take place. One at baseline and thereafter at every step of the intervention (at 3, 6, 9 and 12 months) and at follow-up (at 18 and 24 months). Outcome measures are obtained over the phone by masked research assistants. These assistants are trained (by the first author) to follow a predetermined protocol. The first interview takes approximately 45 minutes; the next interviews take approximately 30 minutes. In Figure 2 an overview of the design is presented.

#### Outcomes

The primary outcome of this study is the incidence of depressive (major depression and dysthymia) or anxiety disorders (panic disorder, agoraphobia, social phobia and generalised anxiety disorder) as measured with the Mini International Neuropsychiatric Interview (MINI) [47], using the electronic version MiniManager 2.0 (de Beurs, Leiden University Medical Centre). Secondary outcome measures are subthreshold depression measured with the Centre for Epidemiologic Studies Depression scale (CES-D) [37,38] and subthreshold anxiety measured with the Hospital Anxiety and Depression Scale – Anxiety (HADS-A) [39]. Other secondary outcome measures are vision related quality of life as measured with the Low Vision Quality Of Life questionnaire (LVQOL) [49,50] and health related quality of life measured with the EuroQol-5 Dimensions (EQ-5D) [51]. Adaptation to vision loss is measured with the Adaptation to age-related Vision Loss (AVL) questionnaire [52] and perceived need for care is measured with the Perceived Need for Care Questionnaire (PNCQ) [53]. For the process-evaluation the Dutch Mental Healthcare Thermometer (DMH thermometer) is used to evaluate the different steps of the stepped-care programme [54]. For the cost-evaluation the Trimbos/iMTA questionnaire for Costs associated with Psychiatric illness (TicP) is used to measure health care utilisation [55] and the Short Form Health and Labour Questionnaire (SF-HLQ) to measure absenteeism and presenteeism from paid and unpaid work [56]. Standard costs for health care utilization from the Dutch costing guidelines are used [57]. Medication use is valued using prices of the Royal Dutch Society of Pharmacy. Productivity losses will be valued using the Friction Cost Approach. In Table 1 the different outcome measures are presented per follow-up measurement.

**Table 1:** Outcome measures, instruments and assessments

**Steps**	**T0**	**T1**	**T2**	**T3**	**T4**	**T5**	**T6**
		**3 m**	**6 m**	**9 m**	**12 m**	**18 m**	**24 m**
**Primary Outcome**							
Depression or anxiety disorder (MINI) [47]	x	x	x	x	x	x	x
**Secondary Outcomes**							
Subthreshold depression (CES-D) [37,38]	x	x	x	x	x	x	x
Subthreshold anxiety (HADS-A) [39]	x	x	x	x	x	x	x
Six item screener [48]	x						
Vision related quality of life (LVQOL) [49,50]	x				x		x
Health related quality of life (EQ5D) [51]	x				x		x
Adaptation to age-related Vision Loss (AVL) [52]	x				x		x
Perceived need for care (PCNQ) [53]	x	x	x	x	x	x	x
Patient satisfaction (DMH thermometer) [54]		x	x	x	x		
Health care utilisation (Tic-P) [55]	x		x		x		x
Health and labour (SFHLQ) [56]	x		x		x		x

#### Sample size

The power calculation is based on the study of van Van ‘t Veer et al. 2009 in which a comparable stepped-care programme was tested in older adults [32]. Results form this study showed that over a period of two years approximately 40% of older adults in the control group, which was not depressed at baseline, became depressed during this study, versus 20% of older adults in the intervention group. Therefore, it is expected that the programme will lead to a reduction of depressive and anxiety disorders of 50% (approximately 20% versus 40%). Based on a power of 85%, a significance level of 0.05 (two-sided) and a drop-out rate of 20%, 115 patients are needed in each group to demonstrate an effect size of 0.50. Approximately 3000 visually impaired patients (of 50 years and older) of one of the rehabilitation organisations, are invited to participate in the screening for this study. We expect that approximately 30% is willing to cooperate, resulting in 900 patients who can be screened for symptoms of depression and anxiety with the CES-D and HADS-A and on depressive or anxiety disorders with the MINI, by means of a telephone interview. This will result in approximately 230 eligible patients.

#### Statistical analysis

The analyses are based on the intention-to-treat principle. Statistical analyses are performed using SPSS 20. First, descriptive statistics of baseline characteristics are compared to check whether randomisation is successful in generating two similar groups. Survival analyses and mixed modelling is then used to compare the treatment and control group. All available data in all measurement cycles are used to estimate the longitudinal trial outcomes at 3, 6, 9, 12, 18 and 24 months after baseline.

For the economic evaluation multiple imputation is used to impute missing data. Bias-corrected and accelerated bootstrapping with 5000 replications is used to estimate 95% confidence intervals around the mean difference in total costs between the treatment and control group. Incremental cost-effectiveness ratios (ICERs) are calculated. Bootstrapping is used to estimate the uncertainty surrounding the ICERs. Cost-effectiveness acceptability curves and net monetary benefits are also estimated.

## Discussion

Depression and anxiety symptoms are common in visually impaired older adults and this elderly group will only grow in the future because of the ageing of the population. It is crucial to treat depressive and anxiety symptoms, because they are the most important predictors of developing a full-blown depressive or anxiety disorder. However, the evidence for preventive interventions has received little attention in the field of low vision. This study aims to investigate an indicated preventive intervention to prevent the onset of depressive and anxiety disorders in patients that show early signs of the disorders.

### Strengths

This study is innovative because the (cost-)effectiveness of a stepped-care programme to prevent depression and anxiety has never been tested in a visually impaired population before. Additionally, the programme is based on successful stepped-care interventions for older adults in the general population [31-33] and other effective components of preventive interventions in the field of low vision [21,24]. Another strength of this study is that the treatment is aimed at preventing both depression and anxiety. The few studies, that investigated interventions to reduce depression in the field of low vision, did not take anxiety into account while several other studies suggest that anxiety is as important a problem for visually impaired older adults as depression is [58,59]. Moreover, this stepped-care programme is embedded within rehabilitation organisations for the visually impaired, where health care workers with the specific expertise of working with visually impaired older adults can offer treatment to reduce symptoms of depression and anxiety in addition to usual visual rehabilitation care. It is very important to address depression and anxiety in this setting because depression and anxiety seriously complicate successful rehabilitation. By letting healthcare workers from the rehabilitation organisations provide the treatment themselves, continuation of the intervention after the end of this trial is highly improved. This pragmatic design enhances the generalisability of this study.

### Challenges

The recruitment of participants is likely to be difficult because it is a challenging subject for a very frail population (older adults, visually impaired and symptoms of depression and/or anxiety). To address this challenge and to allow patients to receive oral information about the study, all patients (approximately N = 3000) who receive written patient information on the study are approached by telephone by the rehabilitation organisations. Still, drop-out rates may be high because of the vulnerability of the population (sickness, death) and because of the quantity and types of questions that are asked. The way in which these questions are asked may be confronting and cause feelings of sadness or worry. Patients might also have low motivation to comply to the different steps of the programme, because of their depressive state [60]. Therapist support will be used to stimulate their motivation [45]. Another challenge is the possibility of contamination: health care workers might give more attention to problems of depression and anxiety because of their newly learned skills to provide support for these problems to patients outside the intervention group. Therefore, it is stressed to health care workers to not offer treatment according to the stepped-care programme to patients outside the intervention group. Finally, it may be difficult to weigh the specific contributions of the various steps in the stepped-care programme, because of the pragmatic design of the study. Not all patients of the intervention group follow the same steps of the programme.

## Conclusion

The importance and strengths of this study outweigh the challenges. The development and research of the (cost-)effectiveness of a stepped-care programme to prevent depressive and anxiety disorders in visually impaired older adults is of the greatest importance.

## Abbreviations

AMD: Age-related macular degeneration; PST: Problem solving treatment; GP: General practitioner; RCT: Randomised controlled trial; CBT: Cognitive behavioural therapy.

## Competing interests

The authors declare that they have no competing interests.

## Authors’ contributions

RvN conceived of the study and its design. GvR, HC, JB and TM advised in the development of the design. HvdA drafted the manuscript, which was revised by the other authors. All authors read and approved the final manuscript.

## Pre-publication history

The pre-publication history for this paper can be accessed here:

http://www.biomedcentral.com/1471-244X/13/209/prepub

## References

[B1] World Health Organization Visual impairment and blindnesshttp://www.who.int/mediacentre/factsheets/fs282/en/

[B2] Limburg H Keunen JE Blindness and low vision in The Netherlands from 2000 to 2020-modeling as a tool for focused intervention Ophthalmic Epidemiol 2009 16 6 362 369 10.3109/09286580903312251 19995201

[B3] Brody BL Gamst AC Williams RA Smith AR Lau PW Dolnak D Rapaport MH Kaplan RM Brown SI Depression, visual acuity, comorbidity, and disability associated with age-related macular degeneration Ophthalmology 2001 108 10 1893 1900 10.1016/S0161-6420(01)00754-0 11581068

[B4] Rovner BW Casten RJ Activity loss and depression in age-related macular degeneration American Journal of Geriatric Psychiatry 2002 10 3 305 310 11994218

[B5] Horowitz A Reinhardt JP Boerner K Travis LA The influence of health, social support quality and rehabilitation on depression among disabled elders Aging Ment Health 2003 7 5 342 350 10.1080/1360786031000150739 12959803

[B6] Horowitz A Reinhardt JP Kennedy GJ Major and subthreshold depression among older adults seeking vision rehabilitation services Am J Geriatr Psychiatry 2005 13 3 180 187 15728748 10.1176/appi.ajgp.13.3.180

[B7] Beekman AT Copeland JR Prince MJ Review of community prevalence of depression in later life Br J Psychiatry 1999 174 307 311 10.1192/bjp.174.4.307 10533549

[B8] Smit F Ederveen A Cuijpers P Deeg D Beekman A Opportunities for cost-effective prevention of late-life depression: an epidemiological approach Arch Gen Psychiatry 2006 63 3 290 296 10.1001/archpsyc.63.3.290 16520434

[B9] Mitchell J Bradley C Quality of life in age-related macular degeneration: a review of the literature Health Qual Life Outcomes 2006 4 97 10.1186/1477-7525-4-97 17184527 PMC1780057

[B10] Huang CQ Dong BR Lu ZC Yue JR Liu QX Chronic diseases and risk for depression in old age: a meta-analysis of published literature Ageing Res Rev 2010 9 2 131 141 10.1016/j.arr.2009.05.005 19524072

[B11] Schutser JP Manetti A Aeschimann M Limosin F **Epidemiology of psychiatric disorder in elderly and their impact on somatic health** Geriatr Psychol Neuropsychiatr Vieil 2013 11 2 181 185 23803635 10.1684/pnv.2013.0405

[B12] Smits F Smits N Schoevers R Deeg D Beekman A Cuijpers P An epidemiological approach to depression prevention in old age Am J Geriatr Psychiatry 2008 16 6 444 453 10.1097/JGP.0b013e3181662ab6 18515688

[B13] Casten RJ Rovner BW Tasman W Age-related macular degeneration and depression: a review of recent research Curr Opin Ophthalmol 2004 15 3 181 183 10.1097/01.icu.0000120710.35941.3f 15118503

[B14] O'Donnell C The Greatest Generation meets its Greatest Challenge: Vision Loss and Depression in Older Adults Journal of visual impairment & blindness 2005 99 197 20823942556

[B15] Cairney J Corna LM Veldhuizen S Herrmann N Streiner DL Comorbid depression and anxiety in later life: patterns of association, subjective well-being, and impairment Am J Geriatr Psychiatry 2008 16 3 201 208 18310551 10.1097/JGP.0b013e3181602a4a

[B16] Kvaal K McDougall FA Brayne C Matthews FE Dewey ME Co-occurrence of anxiety and depressive disorders in a community sample of older people: results from the MRC CFAS (Medical Research Council Cognitive Function and Ageing Study) Int J Geriatr Psychiatry 2008 23 3 229 237 10.1002/gps.1867 17631679

[B17] King-Kallimanis B Gum AM Kohn R Comorbidity of depressive and anxiety disorders for older Americans in the national comorbidity survey-replication Am J Geriatr Psychiatry 2009 17 9 782 792 10.1097/JGP.0b013e3181ad4d17 19700950

[B18] Beekman AT de BE, van Balkom AJ Deeg DJ van DR van TW Anxiety and depression in later life: Co-occurrence and communality of risk factors Am J Psychiatry 2000 157 1 89 95 10618018 10.1176/ajp.157.1.89

[B19] Horowitz A Reinhardt JP Boerner K The effect of rehabilitation on depression among visually disabled older adults Aging Ment Health 2005 9 6 563 570 10.1080/13607860500193500 16214704

[B20] Horowitz A Brennan M Reinhardt J MacMillan T The impact of assistive device use on disability and depression among older adults with age-related vision impairments Journals of gerontology series B-psychological sciences and social sciences 2006 61 5 274 280 10.1093/geronb/61.5.S274 16960241

[B21] Brody BL Roch-Levecq AC Kaplan RM Moutier CY Brown SI Age-related macular degeneration: self-management and reduction of depressive symptoms in a randomized, controlled study J Am Geriatr Soc 2006 54 10 1557 1562 10.1111/j.1532-5415.2006.00881.x 17038074

[B22] Rees G Saw CL Lamoureux EL Keeffe JE Self-management programs for adults with low vision: needs and challenges Patient Educ Couns 2007 69 1–3 39 46 17686604 10.1016/j.pec.2007.06.016

[B23] Girdler SJ Boldy DP Dhaliwal SS Crowley M Packer TL Vision self-management for older adults: a randomised controlled trial Br J Ophthalmol 2010 94 2 223 228 10.1136/bjo.2008.147538 20139291

[B24] Rovner BW Casten RJ Preventing late-life depression in age-related macular degeneration Am J Geriatr Psychiatry 2008 16 6 454 459 10.1097/JGP.0b013e31816b7342 18515689 PMC3591459

[B25] Rovner BW Casten RJ Hegel MT Massof RW Leiby BE Tasman WS Improving function in age-related macular degeneration: design and methods of a randomized clinical trial Contemp Clin Trials 2011 32 2 196 203 10.1016/j.cct.2010.10.008 20974293 PMC3034775

[B26] Margrain TH Nollett C Shearn J Stanford M Edwards RT Ryan B Bunce C Casten R Hegel MT Smith DJ The Depression in Visual Impairment Trial (DEPVIT): trial design and protocol BMC Psychiatry 2012 12 1 57 10.1186/1471-244X-12-57 22672253 PMC3395562

[B27] Haaga DA Introduction to the special section on stepped care models in psychotherapy J Consult Clin Psychol 2000 68 4 547 548 10965628

[B28] Araya R Rojas G Fritsch R Gaete J Rojas M Simon G Peters TJ Treating depression in primary care in low-income women in Santiago, Chile: a randomised controlled trial Lancet 2003 361 9362 995 1000 10.1016/S0140-6736(03)12825-5 12660056

[B29] Davidson KW Rieckmann N Clemow L Schwartz JE Shimbo D Medina V Albanese G Kronish I Hegel M Burg MM Enhanced depression care for patients with acute coronary syndrome and persistent depressive symptoms: coronary psychosocial evaluation studies randomized controlled trial Arch Intern Med 2010 170 7 600 608 10.1001/archinternmed.2010.29 20386003 PMC2882253

[B30] Patel V Weiss HA Chowdhary N Naik S Pednekar S Chatterjee S De Silva MJ Bhat B Araya R King M Simon G Verdeli H Kirkwood BR Effectiveness of an intervention led by lay health counsellors for depressive and anxiety disorders in primary care in Goa, India (MANAS): a cluster randomised controlled trial Lancet 2010 376 9758 2086 2095 10.1016/S0140-6736(10)61508-5 21159375 PMC4964905

[B31] Unutzer J Katon WJ Fan MY Schoenbaum MC Lin EH La Penna RD Powers D Long-term cost effects of collaborative care for late-life depression Am J Manag Care 2008 14 2 95 100 18269305 PMC3810022

[B32] van't Veer-Tazelaar PJ Van Marwijk HWJ Van Oppen P Van Hout HPJ van der Horst HE Cuijpers P Smit F Beekman ATF Stepped-care prevention of anxiety and depression in late life: a randomized controlled trial Arch Gen Psychiatry 2009 66 3 297 304 10.1001/archgenpsychiatry.2008.555 19255379

[B33] van't Veer-Tazelaar PJ Van Marwijk HWJ Van Oppen P van der Horst HE Smit F Cuijpers P Beekman ATF Prevention of late-life anxiety and depression has sustained effects over 24 months: a pragmatic randomized trial Am J Geriatr Psychiatry 2011 19 3 230 239 10.1097/JGP.0b013e3181faee4d 21425519

[B34] Balkom ALJM Hermens MLM Emmelkamp PMG Vliet IM Meeuwissen JAC Bockting CLH Spijker J Multidisciplinaire richtlijn Depressie (Tweede revisie). Richtlijn voor de diagnostiek, behandeling en begeleiding van volwassen patiënten met een depressieve stoornis 2012 Utrecht: Trimbos Instituut http://www.ggzrichtlijnen.nl/

[B35] National Institute for Health and Clinical Excellence (NICE) Depression in adults with a chronic physical health problemhttp://publications.nice.org.uk/depression-in-adults-with-a-chronic-physical-health-problem-cg91

[B36] Beekman A Mihalopoulos C Smit F Van SA Cuijpers P Preventing the onset of depressive disorders: a meta-analytic review of psychological interventions Am J Psychiatry 2008 165 10 1272 1280 10.1176/appi.ajp.2008.07091422 18765483

[B37] Breslau N Depressive symptoms, major depression, and generalized anxiety: a comparison of self-reports on CES-D and results from diagnostic interviews Psychiatry Res 1985 15 3 219 229 10.1016/0165-1781(85)90079-4 3862157

[B38] Beekman AT Deeg DJ Van LJ Braam AW De Vries MZ van TW Criterion validity of the Center for Epidemiologic Studies Depression scale (CES-D): results from a community-based sample of older subjects in The Netherlands Psychol Med 1997 27 1 231 235 10.1017/S0033291796003510 9122304

[B39] Snaith RP Zigmond AS The hospital anxiety and depression scale Br Med J (Clin Res Ed) 1986 292 6516 344 10.1136/bmj.292.6516.344 PMC1339318 3080166

[B40] Barrett JE Williams JW Jr Oxman TE Frank E Katon W Sullivan M Hegel MT Cornell JE Sengupta AS Treatment of dysthymia and minor depression in primary care: a randomized trial in patients aged 18 to 59 years J Fam Pract 2001 50 5 405 412 11350703

[B41] Lynch D Tamburrino M Nagel R Smith MK Telephone-based treatment for family practice patients with mild depression Psychol Rep 2004 94 3 Pt 1 785 792 15217028 10.2466/pr0.94.3.785-792

[B42] Hegel MT Oxman TE Hull JG Swain K Swick H Watchful waiting for minor depression in primary care: remission rates and predictors of improvement Gen Hosp Psychiatry 2006 28 3 205 212 10.1016/j.genhosppsych.2006.02.008 16675363

[B43] Cuijpers P Psychological outreach programmes for the depressed elderly: a meta-analysis of effects and dropout Int J Geriatr Psychiatry 1998 13 1 41 48 10.1002/(SICI)1099-1166(199801)13:1<41::AID-GPS729>3.0.CO;2-B 9489580

[B44] Kühner C Das Gruppenprogramm 'Depression bewaeltigen' und seine Varianten - eine aktualisierte Verhaltenstherapie 2003 13 254 262 10.1159/000075841

[B45] Miller WR Rollnick S Motiverende gespreksvoering 2005 Gorinchem: Ekklesia

[B46] Nieuwsma JA Trivedi RB McDuffie J Kronish I Benjamin D Williams JW Brief psychotherapy for depression: a systematic review and meta-analysis Int J Psychiatry Med 2012 43 2 129 151 10.2190/PM.43.2.c 22849036 PMC3668561

[B47] Sheehan DV Lecrubier Y Sheehan KH Amorim P Janavs J Weiller E Hergueta T Baker R Dunbar GC The Mini-International Neuropsychiatric Interview (M.I.N.I.): the development and validation of a structured diagnostic psychiatric interview for DSM-IV and ICD-10 J Clin Psychiatry 1998 59 22 33 Suppl 20 9881538

[B48] Callahan CM Unverzagt FW Hui SL Perkins AJ Hendrie HC Six-item screener to identify cognitive impairment among potential subjects for clinical research Med Care 2002 40 9 771 781 10.1097/00005650-200209000-00007 12218768

[B49] Wolffsohn JS Cochrane AL Design of the low vision quality-of-life questionnaire (LVQOL) and measuring the outcome of low-vision rehabilitation Am J Ophthalmol 2000 130 6 793 802 10.1016/S0002-9394(00)00610-3 11124300

[B50] Van Nispen RM Knol DL Neve HJ Van Rens GH A multilevel item response theory model was investigated for longitudinal vision-related quality-of-life data J Clin Epidemiol 2010 63 3 321 330 10.1016/j.jclinepi.2009.06.012 19766455

[B51] EuroQol group Euroqol -- a new facility for the measurement of health-related quality of life. The EuroQol Group Health Policy 1990 16 3 199 208 10109801 10.1016/0168-8510(90)90421-9

[B52] Horowitz ARJ Raykov T Development and validation of a short-form adaptation of the Age-related Vision Loss scale: The AVL12 Journal Visual Impairment Blindness 2013 3 146 159

[B53] Meadows G Harvey C Fossey E Burgess P Assessing perceived need for mental health care in a community survey: development of the Perceived Need for Care Questionnaire (PNCQ) Soc Psychiatry Psychiatr Epidemiol 2000 35 9 427 435 10.1007/s001270050260 11089671

[B54] Kok I Van Wijngaarden B Client Appreciation in Mental Health Care: Manual of the Dutch Mental Healthcare Thermometer of Appreciation by Clients 2003 Utrecht: Trimbos-instituut/GGZ Nederland

[B55] Hakkaart-van Roijen L Manual Trimbos/iMTA questionnaire for Costs associated with Psychiatric Illness (TiC-P). Institute for Medical Technology Assessment 2002 Rotterdam: Erasmus University Medical Centre

[B56] Hakkaart-van Roijen L Bouwmans CAM Manual Short Form-Health and Labour Questionnaire. Institute for MTA 2002 Rotterdam: Erasmus University Medical Centre

[B57] Hakkaart-van Roijen L Tan SS Bouwmans CAM Dutch manual for costing in economic evaluations 2011 Diemen: College Voor Zorgverzekeringen (CVZ) 10.1017/S0266462312000062 22559757

[B58] Augustin A Sahel JA Bandello F Dardennes R Maurel F Negrini C Hieke K Berdeaux G Anxiety and depression prevalence rates in age-related macular degeneration Invest Ophthalmol Vis Sci 2007 48 4 1498 1503 10.1167/iovs.06-0761 17389477

[B59] Soubrane G Cruess A Lotery A Pauleikhoff D Mones J Xu X Zlateva G Buggage R Conlon J Goss TF Burden and health care resource utilization in neovascular age-related macular degeneration: findings of a multicountry study Arch Ophthalmol 2007 125 9 1249 1254 10.1001/archopht.125.9.1249 17846366

[B60] MacLeod M Martinez R Williams C Cognitive Behaviour Therapy self-help: who does it help and what are its drawbacks? Behav Cogn Psychother 2009 37 1 61 72 10.1017/S1352465808005031 19364408

